# School-going adolescent girls’ preferences and views of family planning services in Phalombe district, Malawi: A descriptive, cross-sectional study

**DOI:** 10.1371/journal.pone.0267603

**Published:** 2022-05-03

**Authors:** Chancy Skenard Chimatiro, Felistas Mpachika-Mfipa, Lumbani Tshotetsi, Precious L. Hajison

**Affiliations:** 1 Department of Administration, Phalombe District Health Office, Phalombe, Malawi; 2 Department of Nursing, Phalombe District Health Office, Phalombe, Malawi; 3 Clinical Associate Program (Family Medicine), University of Pretoria, Pretoria, Hatfield, South Africa; 4 Preluha Consultancy, Zomba, Malawi; Freelance Consultant, Myanmar, MYANMAR

## Abstract

**Background:**

Low uptake of family planning services by adolescent girls remains a public health concern. An estimated 120 out of every 1,000 girls aged 15 to 19 years are having unplanned pregnancies in the sub-Saharan region. Between January and June 2020, the Phalombe District of Malawi reported 3,030 adolescent pregnancies. At this stage, most Malawian schools were closed due to the COVID-19 pandemic. The high rate of adolescent pregnancies prompted the Ministry of Health to provide emergency contraceptives to reduce the number of unplanned pregnancies among adolescents. The provision of emergency contraceptives would be effective if girls were willing and able to access these family planning services. We thus explored the views of school-going adolescent girls regarding their preferences for modern family planning methods including emergency contraceptives in Phalombe, Malawi.

**Methods:**

This was a cross-sectional, descriptive study, where quantitative data were collected using a structured questionnaire. Participants included randomly sampled school-going adolescent girls from eight purposively selected secondary schools and eight randomly selected primary schools. All the schools were sampled from three purposively selected Traditional Authorities namely Nkhulambe, Jenala and Nkhumba which had reported high numbers of adolescent pregnancies. We analyzed the GeoPoints for schools and health facilities using ArcGIS, while adolescent girls’ views were analyzed using STATA.

**Results:**

Participants included 388 adolescent girls, ranging in age from 10 to 19 years (median age = 15.5 years, SD = 1.9 years). Participants were hesitant to use contraceptives because they were afraid of being stigmatized and embarrassed, had to travel long distances to reach the service center, knew little about modern family planning and were afraid of medical complications.

**Conclusion:**

The uptake of family planning services by adolescent girls can be improved by bringing healthcare services closer to schools and homes. Family planning services should employ health workers who are non-judgmental and who are able to remove the stigma associated with family planning. Health workers should at any given opportunity, address the misconceptions and beliefs that adolescents have towards contraceptives. Community sensitization and health talks should be done to improve adolescent girls’ understanding of family planning services.

## Introduction

Low uptake of family planning methods in adolescents remains a public health concern. An estimated 120 out of every 1,000 girls aged 15 to 19 years have experienced an unplanned pregnancy in the sub-Saharan region [1]. Unplanned pregnancies in adolescent girls often lead to unsafe abortions and school drop-out [2]. Adolescent girls are also at higher risk of maternal mortality. In Zomba, Malawi, researchers explored the risk factors of teenage pregnancy amongst 505 adolescent girls and reported that more than 76% of pregnancies were unplanned [3].

To address the social burden of teenage pregnancy, the World Health Organization (WHO) recommends strengthening sexual and reproductive health services among adolescent girls [4]. These services include long and short-term family planning methods. Emergency contraceptives (ECs) fall in the short-term category of family planning methods. The WHO defines ECs as contraception that women of reproductive age may use following unprotected sexual intercourse, contraceptive failure, incorrect contraceptive use or sexual assault [5]. Emergency contraceptives should be taken within 72 hours after sexual intercourse and can prevent over 95% of pregnancies when taken within this specified period [6]. In principle, efficient use of ECs could prevent most unwanted pregnancies.

In Malawi, modern family planning including ECs are available but are not readily accessible or used by adolescents. The 2015–16 Malawi Demographics and Health Survey indicated that only 45% of women across all child bearing age groups used ECs [7]. The use of modern family planning methods including ECs is much lower in adolescent girls hence the high prevalence of unplanned pregnancies amongst teenagers [8]. In Nigeria, adolescent girls had high levels of knowledge and positive attitudes towards ECs but the use of ECs remained low [9, 10]. Many factors are associated with low use of contraceptives in adolescents, including fear of others’ and parental attitudes, availability and accessibility of contraceptives, and peer influence [11, 12].

Adolescent girls have unique emotional and physical needs, which need to be considered if they are to access family planning methods including ECs. Certain factors may help adolescent girls to access contraceptives easily [13]. For example, in the Ntcheu district of Malawi, young people preferred to receive contraceptives from friendly and non-judgmental service providers [14]. Most adolescent girls also prefer to access and use family planning including ECs in settings where confidentiality and privacy are observed, without having to incur transport costs [15].

In 2020, the Malawian Ministry of Health prioritized the provision of ECs in the Phalombe district to reduce the incidence of unplanned pregnancies among adolescent girls. Between January and June 2020, when schools were closed due to the first wave of the COVID-19 pandemic, 3,030 adolescent pregnancies were reported in the Phalombe district [16, 17].

Adolescent girls should be encouraged to use modern family planning methods including ECs, especially in low and middle-income countries (LMICs) [9]. Contraceptive use is a culturally sensitive issue and studies investigating perceptions should be contextually relevant. Since ECs were introduced in the Phalombe district of Malawi, no studies have explored adolescent girls’ perceptions of family planning services in the district. In this study, we explore the views of school going adolescent girls and their preferences for modern family planning services including ECs in Phalombe, Malawi.

## Methods and materials

### Study design

In this cross-sectional descriptive study, quantitative data were collected using a structured data collection tool.

### Participant identification and recruitment

The study population included all school going adolescent girls aged between the ages of 10 and 19 years. We purposively selected three traditional authorities (TAs) located in the Phalombe district of Malawi. Traditional authorities are represented by local leaders who are gate keepers and influential in safeguarding cultural norms. These local leaders are also involved in promoting the sexual and reproductive health rights of girl-children by creating bye-laws to protect adolescent girls. Adolescent girls were selected from TAs where numerous adolescent pregnancies had been reported between January and June 2020 when schools were closed due to the COVID-19 pandemic. We identified eight secondary schools in the TAs, which were all purposively included in the study. We also randomly selected eight primary schools in the TAs. From the selected schools, we purposively selected classes in levels five to eight (primary) and nine to 12 (secondary). These were senior classes where most adolescents aged 10 to 19 years were expected to be found. We randomly selected six adolescent girls from each class to achieve a representative sample. Each class was thus treated independently. If a selected participant, or caregiver, refused consent, we replaced the participant by randomly selecting the next available participant. We used cluster sampling, and 2 strata were assumed, namely primary school going girls and secondary school going girls. We calculated sample size as 6% of adolescent girls who accessed family planning in the Phalombe district during 2020 according to the HMIS report. We used 5% marginal error, and design factor of 2. We assumed a 90% response rate and 95% power. We calculated a sample size of 388 participants, 194 each from primary and secondary schools. The study was conducted between April and June 2021.

### Study site

The study was conducted in three TAs of Phalombe District, Malawi. The three TAs sampled included Jenala, Nkhumba and Nkhulambe. The Phalombe district lies in the South-East part of Malawi and shares boundaries with Mulanje to the south and west, Zomba to the north and Mozambique to the east. Most inhabitants belong to the Lomwe tribe who depend on domestic or subsistence farming.

### Data collection

Data were collected by five research assistants, who completed a five-day training course on how to use the data collection tool, which was a structured questionnaire. Before collecting data, we pretested and validated the questionnaire. After the pretest, we rephrased questions that were difficult to ask and understand. Data were collected during face to face interviews, where the research assistants recorded the answers given by the participants. All COVID-19 protocols were observed by ensuring that the interviewers and the study participants wore face masks and that physical distancing was observed. After completing each interview, research assistants double checked that all the questions were properly completed before the participant left. Research assistants also shared the completed questionnaires for members to check for common errors. The supervisor conducted a final check of each questionnaire and returned the questionnaire to the research assistant if inconsistencies and missing values were noticed. Research assistants then returned to participants and updated the questionnaires to include the correct information.

### Ethics

Data were collected in accordance with the principles of the Declaration of Helsinki. The protocol was approved by the National Health Sciences Research Committee (NHSRC), Lilongwe, Malawi (#2l/0312668). The District Commissioner for Phalombe district granted permission to collect data. We obtained written informed consent from the legal guardians of all girls younger than 18 years old. We also obtained assent from the participating girls who were younger than 18 years old. All participants who were 18 and 19 years old were asked to consent on their own and sign written informed consent forms. We assured the participants that participation was voluntary, anonymous, private and confidential. All participants agreed to the publication of results.

### Data management

All data were captured and entered in an Epidata® Database. We designed a template to limit the capturing of impossible data values, and all skip patterns were built in. Data were double entered to eliminate possible data entry errors. Where data were entered differently, we cross-checked the original questionnaire and corrected errors. Data were exported to STATA and cleaned before analysis. All incomplete and outlier values were checked against the original questionnaires before being classified as missing data with a designated code.

### Data analysis

Data were tabulated in STATA^®^, and described using proportions. We also calculated the proportions of the multiple responses to summarize the views and preference of adolescents as to how modern family planning services including ECs are or should be delivered.

We generated maps to show the locations of schools that were sampled in relation to the locations of all schools in the Phalombe district. We also mapped the locations of all public health facilities to assess their proximity to schools. We quantified access to health facilities using a 5km buffer zone around health facilities. We assumed that 5km’s was the maximum distance that students could comfortably travel from school to access health services. All the schools that fell outside the health facility buffer zones were considered to not have easy access to health facilities.

## Results

### Demographic characteristics

A total of 388 adolescent girls participated in the study, ranging in age from 10 to 19 years old (median age = 15.5 years, SD = 1.9). Participants were almost equally distributed across education levels from level 5 to level 12, with levels 5 to 8 being primary and 9 to 12 being secondary schools. The median school level was 8.5 years (SD = 2.3).

The demographic characteristics of the girls are summarized in **Table 1**. Almost half of the participants were in secondary school (50.2%). Most of the adolescents belonged to Pentecostal churches (54.13%), followed by Catholic (17.53%) and Muslims were the fewest with only 2.8%. Most girls belonged to the Lomwe tribe (93.56%). More than 26.55% (n = 103) of adolescent girls were sexually active, acknowledging that they had sex before the study date.

**Table 1:** Demographic characteristics of the study participants.

Variable	Sample Size (n = 388)	Frequency %
Religion		
Muslim	11	2.84
Catholic	68	17.53
CCAP*	46	11.86
Anglican	5	1.29
Seventh day Adventist	48	12.37
Pentecostal churches	210	54.13
Education level		
Primary	193	49.74
Secondary	195	50.26
Tribe		
Chewa	13	3.35
Lomwe	363	93.56
Yao	3	0.77
Mang’anja	3	0.77
Others	6	1.55
Ever had sex before		
Yes	103	26.55
No	285	73.45

CCAP*: Church of Central African Presbyterian.

### Knowledge about ECs and other modern contraceptives

**Fig 1:**
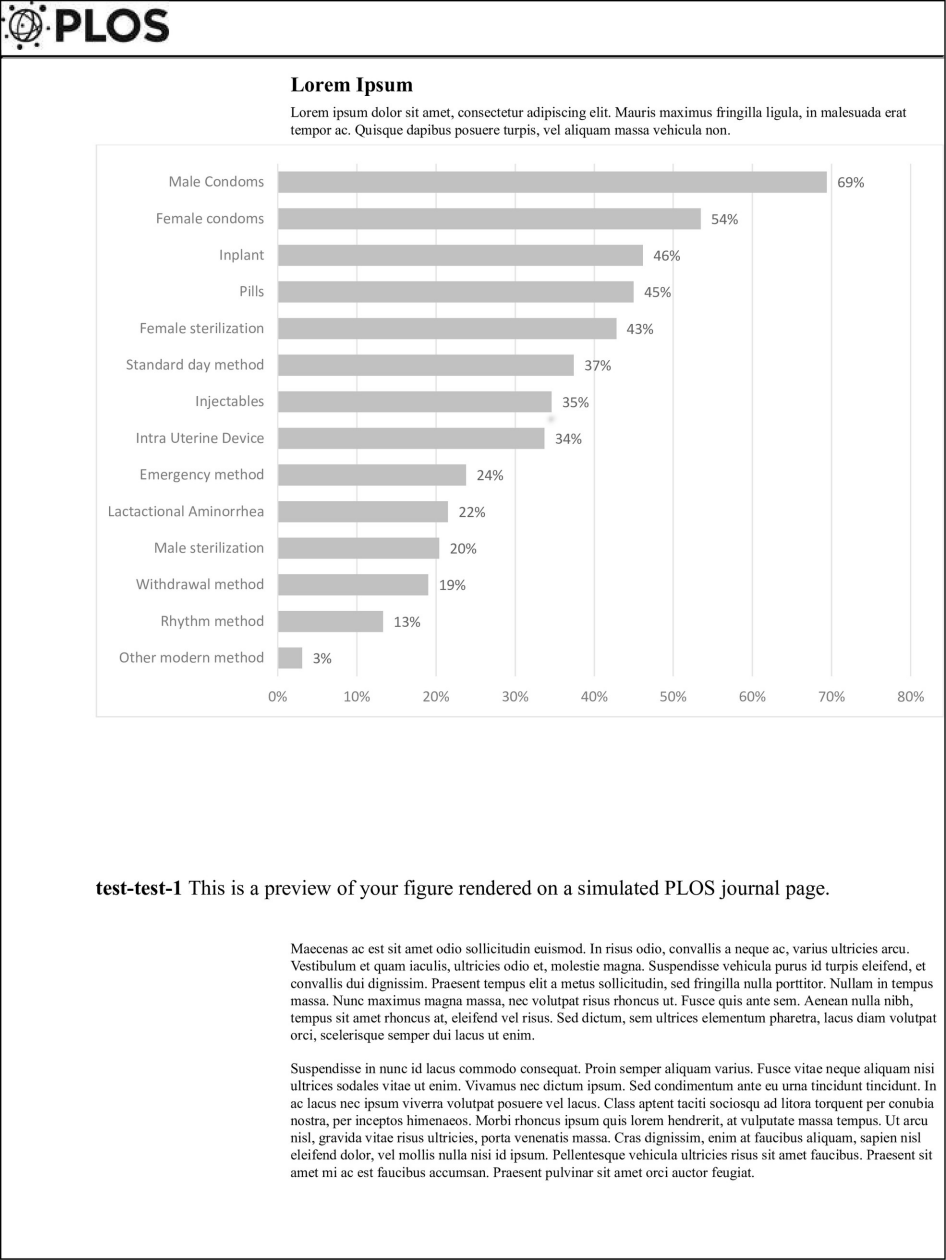
Adolescent girls’ knowledge about available modern family planning in the Phalombe district of Malawi. The figure presents awareness of different family planning methods among adolescents in Phalombe District of Malawi.

Adolescent girls’ knowledge of existing contraceptives is displayed in **Fig 1**. To assess knowledge level, research assistants asked girls if they had ever heard about any of the mentioned methods of family planning. In general, 82.2% (n = 319) of girls knew about at least one method of family planning. Only 24% (n = 93) of girls had ever heard about ECs, while most girls had heard about male condoms 69% (n = 268). The least known method was the rhythm method or natural family planning method, with only 13% (n = 50) of girls reporting that they had heard of this method (**Fig 1**).

### Use and availability of modern family planning including ECs

We also asked girls if they were using or had used family planning services including ECs and also if they had access to family planning at their location (Table **2**). Only 18.3% (n = 71) of girls had ever used contraceptives. Of these 71, only 2.8% (n = 2) had ever used ECs. Of the girls who used contraceptives, 80.3% (n = 57) mainly used condoms (male or female) as their preferred contraceptive, with male condoms being the most widely used (93%, n = 53). Very few school going adolescent girls in Phalombe indicated using long-term methods such as implants 1.4% (n = 1).

**Table 2:** Usage and availability of modern family planning services including ECs to school going adolescent girls in Phalombe district.

Variable Name		Frequency	Percent (%)
Ever used contraceptive (n = 388)	No	317	81.7
	Yes	71	18.3
Method used for protection from getting pregnant (n = 388)	Abstinence	302	77.8
	Condoms	57	14.7
	Others	29	7.5
Type of family planning method used (n = 71)	Emergency contraceptives	2	2.8
	Condom	57	80.3
	Injection	5	7.0
	Implant	1	1.4
	COC* (Pills)	2	2.8
	STD** day method	4	5.6
Type of condom used (n = 57)	Female condom	4	7.0
	Male condom	53	93.0
Where adolescents get contraceptives (n = 71)	Hospital	35	49.3
	Health centre	3	4.2
	Community distributor	12	16.9
	Friends	7	9.9
	Retail	14	19.7
	HSA***	1	1.4
	Sexual partner	19	26.7
	Both	13	19.1
Availability of modern family planning including EC in the area (n = 388)	Yes	114	29.4
	No	221	57.0
	Don’t know	53	13.7
Duration for adolescent to get family planning method including EC (n = 388)	30 minutes	88	22.7
	1–2 hours	110	28.4
	3 hours and above	190	48.9

COC* = Combined Oral Contraceptives.

STD** = Standard Day Method. It is a fertility awareness-based method of family planning that identifies a fixed set of days in each menstrual cycle when a woman can get pregnant if she has unprotected sex.

HSA*** = Health Surveillance Assistant.

In terms of the availability of ECs and family planning in general, 29.4% (n = 114) of girls reported that ECs were available. Almost half of the girls (48.9%, n = 190) had to travel for longer than three hours to a location where they could access contraceptives. Contraceptives were available within 30 minutes’ travel time for 22.7% (n = 88) of girls, and within one to two hours of travel time for 28.4% of girls.

We mapped the locations for all schools in the district, and linked them to all health facilities in the district. Most of the schools were further than five kilometers from health facilities. In Malawi, health facilities offer family planning services for free.

### Social-cultural beliefs and practices towards family planning in Phalombe district

We also assessed girls’ social-cultural beliefs regarding contraceptive use, and the methods they used to prevent pregnancy (**Table 3**). Almost half of school going adolescent girls believed that using contraceptives before having a child led to infertility (48.0%, n = 181), while others reported that contraceptives are for married women and old people (23.6%, n = 89). Sixteen (4.2%) girls believed that using contraceptives caused prolonged menstrual periods. A few girls believed that pregnancy could be prevented by bathing after sexual intercourse and having sex in water (Table **3**).

**Table 3:** Social and cultural constrains affecting adolescent girls’ use of ECs.

Variable		Frequency	Percent (%)
Ever heard of local medicine used to prevent pregnancy (n 377)	Yes	88	23.3%
	No	289	76.7%
Ever used the local medicine to prevent pregnancy (n 88)	Yes	0	0%
	No	88	100%
Cultural practices and beliefs performed that prevent girls from using ECs (n 377)			
	Use of herbs	7	1.9%
	Contraceptives are for married women and old people	89	23.6%
	Contraceptives before giving birth cause infertility	181	48.0%
	Contraceptives causes long menstruation period	16	4.2%
	People considers you as a prostitute	9	2.4%
	Told during initiation never to use contraceptives	5	1.3%
	Afraid of dying	4	1.1%
	Bathing after sex and make sex in water or having sex while standing	3	0.8%
	Causes cancer/obesity/uterus damage	6	1.6%
	Causes backache	1	0.3%
	Religious belief	5	1.3%
	Don’t know/ never heard	51	13.5%
Social barriers that affect adolescents from getting modern family planning including ECs (n 384)			
	Long distances to the facility	32	8.3%
	Poverty	22	5.7%
	Need to have child	53	13.8%
	Fear and being ashamed	230	59.9%
	Church beliefs	3	0.8%
	Fear of injection/cancer	2	0.5%
	Lack of confidentiality and judgemental attitude of health workers	3	0.8%
	Lack of knowledge/ignorance	5	1.3%
	Unavailability of contraceptive	1	0.3%
	Not having a sexual partner/ lack of interest	2	0.5%
	Parental refusal/peer pressure	5	1.3%
	Never heard/don’t know	26	6.8%

**Fig 2:**
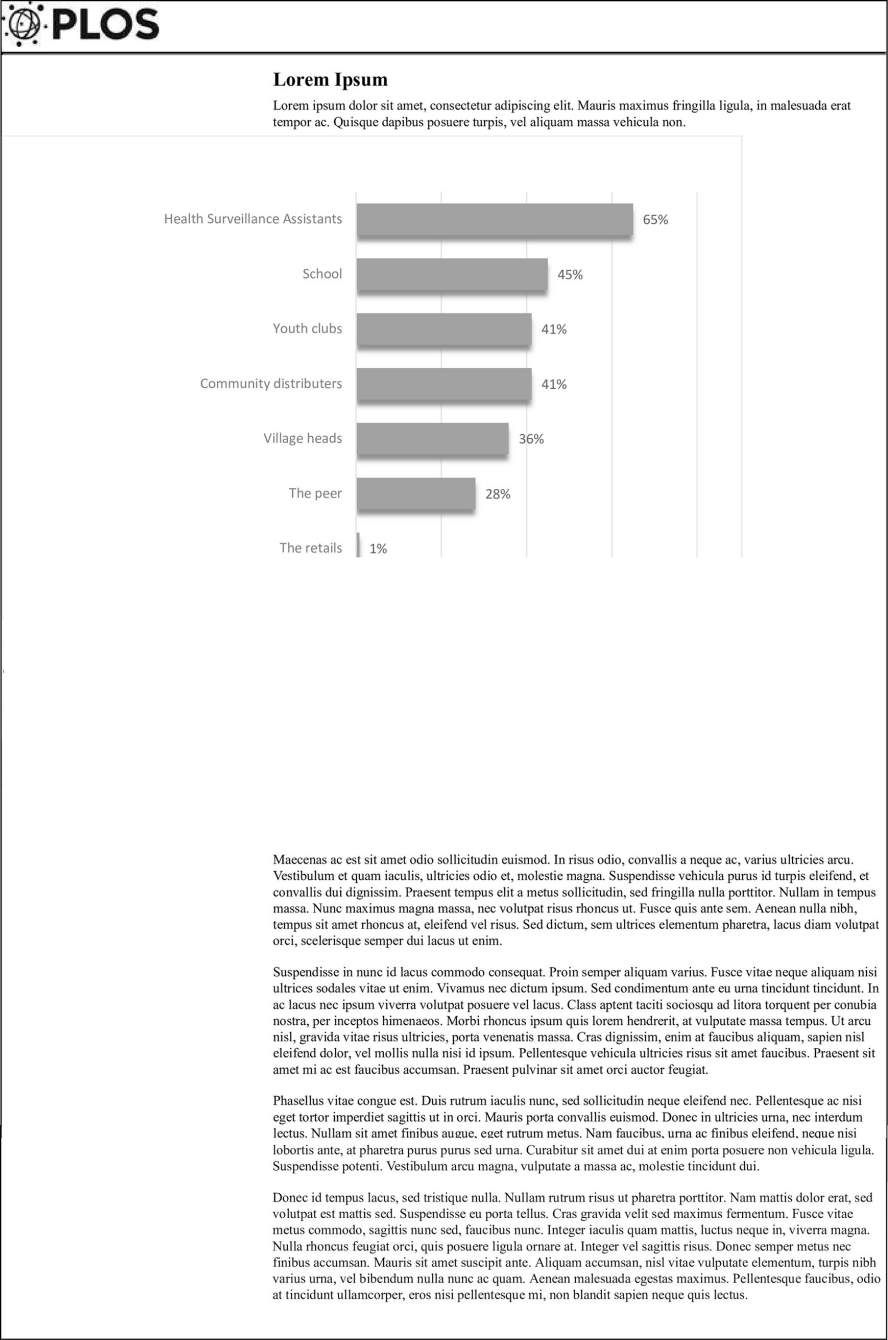
The preferred locations of adolescent girls to access family planning, specifically emergency contraceptives, in the Phalombe district of Malawi. The figure demonstrates opinion of the adolescent’s preference of places and providers where they would want to access family planning services in Phalombe District of Malawi.

Social barriers to contraceptive use by girls included having to travel long distances to health facilities to access ECs (8.3%, n = 32), fear and shame (59.9%, n = 230), need to have child (13.8%, n = 53) and poverty (5.7%, n = 22) (Table **3**). We also asked girls where they would prefer to receive ECs and other family planning services. Most girls (65%) preferred to receive family planning services from Health Surveillance Assistants (HSAs), followed by school and youth clubs and community-based distributors (**Fig 2**).

## Discussion

In this study, we explored factors that limited school going adolescent girls from using family planning methods including ECs in Phalombe district, Malawi. Bridging this gap may reduce many unplanned pregnancies, hence improving adolescent health. In our study, most adolescent girls (82.2%) knew of at least one or more modern family planning methods. Similarly, Damson et al., [18] found that 74% of adolescent girls living in Ntcheu knew about modern contraceptives. In our study, most girls knew about using male condoms for contraception, which was also reported in Ntcheu where 58% of the adolescent girls knew about using condoms as a contraceptive [18]. We suggest that condoms are popular because they are the most advocated mode of contraception after abstinence. In our study, only 24% of adolescent girls knew that ECs existed. Likewise, Babatunde et al., [10] found that few secondary school students in Nigeria knew about ECs, where only 33.3% of participants reported good knowledge. In Nigeria, adolescents who knew little about contraceptives were less likely to use contraceptives [19]. The same may hold true for girls in Malawi, where lack of knowledge is linked to low uptake. School going adolescent girls should be sensitized about the availability of ECs and the importance of using ECs correctly to prevent unwanted pregnancy in this age group.

In our study, a few girls did not know whether family planning was available in their area, but this did not seem to contribute to low usage of contraceptives. Fewer than a quarter of adolescent girls in our study knew about ECs, and only 2.8% had ever used ECs. In Nigeria, adolescents in the Niger Delta Region had high knowledge but low contraceptive use [11], although a larger proportion of adolescent girls (12.8%) had used ECs [11] than reported in our study. The use of ECs and other family planning methods may be determined by factors other than knowledge and availability.

In the Phalombe district, in Malawi, adolescents are guided by community cultural beliefs and norms, which may hinder and limit access to health services and subsequent use. People living in the Phalombe district are living in a rural area, where a large proportion of the population are illiterate. In our study, almost half of girls believed that using contraceptives before having a child leads to infertility. Many girls believed that contraceptives were mainly for married people. A large group of girls were also afraid of being viewed as prostitutes. Similarly, in Ethiopia, adolescents also believed that contraceptives were mainly for adults and they were afraid of being identified or victimized if they sought contraceptives from health care facilities [20]. Adolescents in Nicaragua were also hesitant to use contraceptives because they were afraid of permanent infertility [21]. Many people believe that contraceptives cause a number of health problems including cancer, obesity, uterine damage, backache, menstrual period prolongation and even deaths among adolescent girls. The girls in our study held similar fears and misconceptions about contraceptives which may explain the limited use of modern family planning methods. The limited use of modern family planning methods is evident in the high numbers of unplanned pregnancies reported in the Phalombe district in 2020, when many schools were closed during a COVID-19 lockdown. Previous studies have also reported that adolescent girls believed that contraceptives negatively affect their menstrual cycle [22], similar to the beliefs reported in our study. Young girls therefore require the correct information to clear myths and beliefs surrounding contraceptives to promote the use of family planning methods including ECs. There is an opportunity to change the mind-set of adolescents by providing health talks in schools, churches and other community gatherings.

Aside from knowledge, girls in rural areas may also struggle to access family planning services. In our study, only 22% of girls were able to reach health care services within 30 minutes of their schools, while most girls had travel for three hours or longer to access family planning services including ECs. Most girls in our study would access or accessed family planning services at public health facilities. We found that most schools were further than five kilometers away from health facilities. Girls seeking family planning services would thus have to walk for extended periods to access family planning. It is also likely that the girls in our study lived closer to their schools than to public health facilities, since school attendance is a priority. Distance to health facilities and poverty are known to influence accessibility of health services in Malawi and other LMICs. In Malawi, Chimatiro et al., [23] reported that women who lived further from health facilities were less likely to access early antenatal care. In poor communities, people who live far from health facilities often have transport costs which they cannot afford, resulting in not being able to access health services [24]. In Mozambique, Hayford and Agadjanian [25] reported that travelling time influenced the uptake of family planning services by HIV positive mothers. It is imperative that access to family planning services is improved. In the Phalombe district, satellite clinics could be opened up close enough to schools to ensure easy access, but far enough away to ensure privacy and confidentiality.

The uptake of family planning is a sensitive issue for many adolescent girls, especially in settings where tradition and culture prevents open conversation about sex, especially within households. In our study, most adolescent girls preferred to access family planning from a Health Surveillance Assistant (HSA), which is contrary to current practice where many girls were accessing family planning services at health facilities. In Malawi, the Ministry of Health has deployed HSAs at community level who provide primary health care, including family planning services. Our findings indicate that girls did not know the identities of the HSAs providing family planning services in their locations. In our study, only one girl reported accessing family planning from an HSA. Many girls reported that they would prefer to access family planning services in community-based settings such as schools, youth clubs, community-based distribution agents and village headmen, indicating their trust in these structures. A large barrier to actual use of family planning services in this study was the fear of being embarrassed or stigmatized, should their communities find out that they were using contraceptives [26]. In sub-Saharan Africa, the negative attitudes of health workers have been reported to discourage and prevent adolescents from using family planning methods [27, 28]. In Nigeria, health workers would encourage adolescents to abstain rather than to access family planning, which led to negative attitudes regarding family planning [29]. Health workers also struggled to counsel adolescents because of their personal beliefs [29]. School going adolescent girls in Phalombe preferred more confidential places to access family planning, rather than health facilities. It is also likely that senior health workers are prominent community members, and young girls may be too embarrassed or intimidated to access intimate health care services in such a setting. Girls are afraid of being labelled as prostitutes when they seek contraceptives to protect themselves from unwanted pregnancies. This may be a large barrier preventing most girls from seeking health care even when they need to do so. In central Ghana and Laos, sexually active adolescents seeking contraceptives were stigmatized and perceived as bad or spoilt kids [30–32]. Adolescents are thus shy to access contraceptives including ECs even when they have had unprotected sexual intercourse.

### Study limitations

This is the first study to explore the views of and preference for contraceptives especially ECs among school going adolescent girls in Phalombe, Malawi. Our findings are based on quantitative data and there is still a gap for an in-depth understanding of the challenges faced by adolescents, especially regarding sexual health. This gap can be addressed by conducting a similar study using qualitative methods.

## Conclusion

We explored the views and preference for family planning services among school going adolescent girls in Phalombe district. The uptake of family planning services in this district were influenced by having to travel long distances, fear of health complications such as infertility and being ashamed of using contraceptives. We expect that adolescent girls would prefer to access family planning services closer to their schools, through HSAs who are able to provide trustworthy services in a setting that is comfortable and private. This may encourage girls who would usually shy away from seeking family planning in health facilities where they are more likely encounter judgmental adults. It is imperative that teenage pregnancies are viewed as a public health problem, and that interventions be put in place to sensitize community members and households about the availability and importance of using contraceptives to reduce unplanned pregnancies. Girls should be empowered with knowledge on contraceptives and have access to trustworthy family planning services.

We recommend that adolescents should have access to a wide range of family planning services. Other stakeholders, including parents, teachers and youth community-based distributors, should be supported, so that they can encourage adolescent girls to access modern family planning methods including ECs. Youth friendly services should be encouraged in health facilities and in communities to reduce distress and promote acceptance. We also recommend that out-reach clinics be established closer to schools to reduce the need to travel, especially in rural communities.

## Supporting information

S1 Questionnaire The school going adolescent girls’ preference and views of family planning services questionnaire-English. (PDF)

S2 Questionnaire The school going adolescent girls’ preference and views of family planning services questionnaire-Chichewa. (PDF)

S1 Dataset Dataset used during analysis (DTA). (XLSX)

## References

[1] World Health Organisation. WHO guidelines on preventing early pregnancy and poor reproductive health outcomes among adolescents in developing countries. Geneva: WHO; 2011.

[2] Atuhaire S. Abortion among adolescents in Africa: A review of practices, consequences, and control strategies. Int J Health Plan Man. 2019. 10.1002/hpm.2842 31290183

[3] Kaphagawani NC, Kalipeni E. Sociocultural factors contributing to teenage pregnancy in Zomba district, Malawi. Glob Public Health. 2017;12(6):694–710. doi: 10.1080/17441692.2016.1229354 27687242

[4] WHO recommendations on adolescent sexual and reproductive health and rights. World Health Organisation; 2018.

[5] World Health Organisation. Emergency Contraceptive [Internet]. Fact sheet. 2018. Available from: https://www.who.int/news-room/fact-sheets/detail/emergency-contraception

[6] Emergency Contraception [Internet]. World Health Organisation; 2018 Feb [cited 2021 Aug 13]. Available from: https://www.who.int/news-room/fact-sheets/detail/emergency-contraception

[7] Malawi Demographis and Health Survey 2010. Zomba, Malawi and Calverton, Maryland, USA: NSO and ICF Macro.: National Statistics Office (NSO) and ICF Macro. 2011; 2010.

[8] Ahinkorah BO, Kang M, Perry L, Brooks F, Hellen A. Prevalence of first adolescent pregnancy and its associated factors in sub-Saharan Africa: A multi-country analysis. PLOS ONE 16(2), 2021. doi: 10.1371/journal.pone.0246308 33539394 PMC7861528

[9] Preventing early pregnancy and poor reproductive outcomes among adolescents in developing countries. World Health Organisation; 2011. 10.1016/j.jadohealth.2013.03.002 23608717

[10] Babatunde OA, Ibirongbe DO, Omede O, Babatunde OO. Knowledge and use of emergency contraception among students of public secondary schools in Ilorin, Nigeria. Pan Afr Med J [Internet]. 2016;23(74). 19. Available from: https://www.ncbi.nlm.nih.gov/pmc/articles/PMC4862801/ doi: 10.11604/pamj.2016.23.74.8688 27217897 PMC4862801

[11] Onasoga OA, Afolayan JA, Asamabiriowei TF, Jibril UN. Adolescents’ Knowledge, Attitude and Utilization of Emergency Contraceptive Pills in Nigeria’s Niger Delta Region. Int J MCH AIDS. 2016;5(1):53–60. doi: 10.21106/ijma.93 28058193 PMC5187640

[12] Shiferaw BZ, Gashaw BT, Tesso FY. Factors associated with utilization of emergency contraception among female students in Mizan-Tepi University, South West Ethiopia. BMC Res Notes [Internet]. 2015;8(818). Available from: https://www.ncbi.nlm.nih.gov/pmc/articles/PMC4691018/10.1186/s13104-015-1812-6 PMC4691018 26704070

[13] Seetharaman S, Yen S, Ammerman SD. Improving adolescent knowledge of emergency contraception: challenges and solutions. J Contracept. 2016. doi: 10.2147/OAJC.S97075 29386948 PMC5683156

[14] Michaels-Igbokwe C, Terris-Prestholt F, Chipeta E. Young People’s Preferences for Family Planning Service Providers in Rural Malawi: A Discrete Choice Experiment. Integra Initiat Cairns J [Internet]. 2015;10(12). Available from: 10.1371/journal.pone.0143287 PMC4667908 26630492

[15] Self A, Chipokosa S, Marx MA. Youth accessing reproductive health services in Malawi: drivers, barriers, and suggestions from the perspectives of youth and parents. Reprod Health. 15(108). 10.1186/s12978-018-0549-9 PMC6008927 29921282

[16] UNICEF and its partners are on the ground to respond to COVID-19 in Malawi [Internet]. UNICEF; 2020 Sep [cited 2020 Sep 15]. Available from: https://www.unicef.org/malawi/coronavirus-disease-covid-19

[17] Health Management Information System. Phalombe District Health Office. Phalombe, Malawi; 2020.

[18] Damson E. C., Kabue D., Keraka P. M. Level of Knowledge Among Adolescent Girls On Modern Contraception At Tsangano Turnoff Community, Ntcheu District, Malawi. J Health Med Nurs. 2019;4(6):29–42.

[19] Cortez R, Saadat S, Marinda E, Oluwole O. Adolescent Sexual and Reproductive Health in Nigeria. World Bank Group; 2015.

[20] Birhan Z, Tushune K, Jebena MG. Sexual and reproductive health services use, perceptions, and barriers among young people in southwest Oromia, Ethiopia. Ethiop J Health Sci. 28(1). 10.4314/ejhs.v28i1.6 PMC5866288 29622906

[21] Parker J.J, Veldhuis C, Haider S. Barriers to contraceptive use among adolescents in Nicaragua. Glob Health. 2016. 10.1515/ijamh-2017-0228 30939115

[22] Chernick LS, Schnall R, Higgins T, Stockwell M. Barriers to and Enablers of Contraceptive Use among Adolescent Females and their Interest in an Emergency Department-based Intervention. Contraception. 2015;91(3):217–25. doi: 10.1016/j.contraception.2014.12.003 25499588 PMC4352549

[23] Chimatiro CS, Hajison PL, Chipeta E, Muula A. Understanding barriers preventing pregnant women from starting antenatal clinic in the first trimester of pregnancy in Ntcheu District-Malawi. Reprod Health [Internet]. 2018;15(158). Available from: https://reproductive-health-journal.biomedcentral.com/articles/10.1186/s12978-018-0605-5 30241542 10.1186/s12978-018-0605-5 PMC6151039

[24] Munthali AC, Mannan M, MacLachlan M, Swartz L,. Non-use of Formal Health Services in Malawi: Perceptions from Non-users. Malawi Med J. 2014;26(4):126–32. 26167263 PMC4325348

[25] Hayford SR, Agadjanian V. Providers’ views concerning family planning service delivery to HIV-positive women in Mozambique. Stud Fam Plann. 2010; 41(4): 291–300. doi: 10.1111/j.1728-4465.2010.00254.x 21258608 PMC3023920

[26] Dahab R, Sakellariou D. Barriers to Accessing Maternal Care in Low Income Countries in Africa: A Systematic Review. Int J Env Res Public Health. 2020;17(4292). doi: 10.3390/ijerph17124292 32560132 PMC7344902

[27] Melesse DY, Mutua MK, Choudhury A, Wado YD. Adolescent sexual and reproductive health in sub-Saharan Africa: who is left behind? BMJ-Glob Health [Internet]. 2019; Available from: https://gh.bmj.com/content/bmjgh/5/1/e002231.full.pdf 10.1136/bmjgh-2019-002231 PMC7042602 32133182

[28] Smith J. Improving Adolescent Access to Contraception in Sub-Saharan Africa: A Review of the Evidence. Afr J Reprod Health. 10.29063/ajrh2020/v24i1.16 32358947

[29] Ahanonu EL. Attitudes of Healthcare Providers towards Providing Contraceptives for Unmarried Adolescents in Ibadan, Nigeria. J Family and Reprod Health; 2014; 8(1): https://www.ncbi.nlm.nih.gov/pmc/articles/PMC4064762/ 24971131 PMC4064762

[30] Enuameh Y, Boamah E, Nettey O. Improv Fam Plan Serv Deliv Adolesc Ghana Evid Rural Communities Cent Ghana Meas Eval PRH Work Pap Ser WP-12- 128 USAIDMeasure Eval. 2012.

[31] Boamah EA, Asante KP, Mahama E. Use of contraceptives among adolescents in Kintampo, Ghana: a cross-sectional study. J Contracept. 2014;4(5):7–15.

[32] Thongmixay S, Essink DR, Greeuw T de, Vongxay V. Perceived barriers in accessing sexual and reproductive health services for youth in Lao People’s Democratic Republic. PLoS ONE [Internet]. 2019;14(10). Available from: doi: 10.1371/journal.pone.0218296 31661486 PMC6818758

