# Supplementary material for: School-going adolescent girls’ preferences and views of family planning services in Phalombe district, Malawi: A descriptive, cross-sectional study
Source: PLoS One. 2022 May 3;17(5):e0267603. doi: 10.1371/journal.pone.0267603 (PMC9064102; doi:10.1371/journal.pone.0267603)
Supplement: S1 Questionnaire — (PDF) [file pone.0267603.s001.pdf]

ENGLISH QUESTIONNIERE ON SCHOOL GOING ADOLESCENT GIRLS' PREFERENCE AND VIEWS OF FAMILY PLANNING SERVICES

TIME STARTED:……………………………….

TIME ENDED…………………………………… **DATE:…………………………..**

INTERVIEWER NO ……………………………… **SERIAL NO ……………………**

NO	QUESTIONS	CODING CATEGORIES	SKIP PATTERNS
**PART A: SOCIAL DEMOGRPHIC OF THE RESPONDENTS**<br>**NOW I WOULD LIKE TO ASK SOME QUESTIONS ABOUT YOURSEL**			
01	How old are you?	10-14 (SPECIFY). . . . . 1<br>15-19 (SPECIFY) 2	
02	In which class/form are you?	STANDARD 5-8(SPECIFY). . . . . .1<br>FORM 1-4 (SPECIFY) 2	
03	What is your religion?	MUSLIM . . . . .1<br>CATHOLIC . . . . .2<br>CCAP . . . . .3<br>ANGLICAN . . . . .4<br>SEVETH DAY ADVENTIST/BAPTIST . . . . 5<br>OTHERS CHRISTIAN . . . . 6<br>OTHERS 7	
04	What is your tribe or ethnic group?	CHEWA . . . . .1<br>LOMWE . . . . 2<br>YAO . . . . 3<br>MANG,ANJA . . . . 4<br>OTHERS SPECIFY 5	

PART B-1: KNOWLEDGE ABOUT CONTRACEPTIVES			
05	Have you ever heard or know about contraceptives of any kind?	YES . . . . .1<br>NO 2	→ Q7
06	Which method you ever heard or know?	FEMALE STERILIZATION . . . . A<br>MALE STERILIZATION .B<br>IUD . . . . .C<br>INJECTABLES . . . . .D<br>IMPLANTS . . . . .E<br>PILL . . . . F<br>CONDOM . . .. .G<br>FEMALE CONDOM . . . . .H<br>EMERGENCY CONTRACEPTION . . . . .I<br>STANDARD DAYS METHOD . . . . J<br>LACTATIONAL AMENORRHEA METHOD . . . K<br>RHYTHM METHOD . . . . L<br>WITHDRAWAL . . . . M<br>OTHER MODERN METHOD . . . . N<br>OTHER TRADITIONAL METHOD .. . . O	⟼ Q9
07	Have you ever heard of (PROBE EACH)?	FEMALE STERILIZATION . . . . A<br>MALE STERILIZATION . . . . B<br>IUD C	IF EC NOT MENTIONED GO Q 9

		INJECTABLES . . . . D<br>IMPLANTS . . . . .E<br>PILL . . . . F<br>CONDOM . . . . .G<br>FEMALE CONDOM . . . . H<br>EMERGENCY CONTRACEPTION . . . . .I<br>STANDARD DAYS METHOD . . . . J<br>LACTATIONAL AMENORRHEA METHOD. . . K<br>RHYTHM METHOD.. . . . . L<br>WITHDRAWAL . . . .M<br>OTHER MODERN METHOD . . . . .N<br>EMERGENCE CONTRACEPTIVES . . . . .O	↦ Q9
08	Where did you heard information about EC	GOVERNMENT FACILITIES . . . . 1<br>CHAM . . . . 2<br>PRIVATE HOSPITAL/CLINIC . . . . 3<br>BLM/PSI/GAIA……………. . . . . 4<br>SCHOOL…………………………... . . . . .5<br>FRIENDS……………………….. . . . . 6<br>CHURCH……………………... . . . . 7<br>RADIO (SPECIFY)……………….. . . . . .8<br>TV (SPECIFY)………………………………. . . . 9<br>OTHERS (SPECIFY)………………... 10	
09	Do you know any method that a woman can use to prevent getting pregnancy after having unprotected sex intercourse?	YES . . . . 1<br>NO 2	↦Q11
10	What method is that?	ABSTENANCE 1	

		USING HERBS . . . . 2<br>PILLS (SPECIFY) . . . . .3<br>OTHERS (SPECIFY). 4	
11	What do you do to avoid getting pregnancy	USING HERBS . . . . .1<br>ABSTENIENCE . . . . .2<br>CONTRACEPTIVES . . . . .3<br>OTHERS SPECIFY 4	IF EC MENTIONED ASK Q12 IF NOT GO Q13
12	When can one take EC? ASK RESPONDENT TO SPECIFY	IMMEDIATELY AFTER SEX INTERCOURSE. . . . . .1<br><br>HOURS BEFORE HAVING SEX INTERCOURSE. . . . . .2<br><br>FEW DAYS AFTER HAVING SEXUAL INTERCOIURSE . . . . .3<br><br>DON'T KNOW 4	
13	If you wish today can you easily get EC	YES . . . . .1<br>NO 2	⟼ PART B
14	Where can you get EC (PROBE WHERE ELSE UNTIL NO MORE ANSWER)	GOVERNMENT FACILITIES . . . . .1<br>CHAM . . . . .2<br>PRIVATE HOSPITAL/CLINIC . . . . .3<br>BLM/PSI/GAIA……………. . . . . .4<br>SCHOOL…………………………... . . . . 5<br>FRIENDS……………………….. . . . . 6<br>COMMUNITY DISTRIBITOR. . . . . 7<br>OTHERS (SPECIFY)………………... 8	
	**PART B-2: KNOWLEDGE ABOUT FETILE PERIOD**<br><br>NOW LETS TALK ABOUT WHEN A WOMEN IS MORE LIKELY TO GET PREGNANCY. PLEASE FEEL FREE TO ANSWER OR NOT.		
15	When was your last normal menstrual period?	DAYS AGO . . . . .1<br><br>WEEKS AGO . . . . .2<br><br>MONTHS AGO (SPECIFY) . . . . .3<br><br>NEVER HAD HAD PERIODS 4	⟼17<br><br>⟼17<br><br><br><br>⟼17
16	You have said you experience your last normal menstrual period months ago why?	AM PREGNANT . . . . 1<br>HAVING ABNORNAL PERIODS . . . . 2<br>USING HERBS . . . . 3<br>USING CONTRACEPTIVES . . . . 4<br>DON'T KNOW . . . . 5<br>OTHERS SPECIFY 6	⟼ CHECK PART A ABOVE AND CONFIRM

17	From one menstrual period to the next, are there certain days when a woman is more likely to become pregnancy if she has sexual relations?	YES . . . . 1<br><br>NO . . . . .2<br><br>DON'T KNOW 3	 <br>⟼ GO TO PART C<br><br>⟼ GO TO PART C
18	Is this just before her period begins, during her period, right after her period has ended, or half way between two periods?	JUST BEFORE HER PERIODS BEGINS . . . . . . .1<br><br>DURING HER PERIODS . . . . 2<br><br>RIGHT AFTER HER PERIOD HAS ENDED . . . 3<br><br>HALF WAY BETWEEN TWO PERIODS 4	
	**PART C: AVAILABILITY**<br><br>**NOW I WOULD LIKE TO TALK ABOUT HOW YOU PROTECT YOURSELF FROM GETTING PREGNANCY**		
19	In past six months have you used any pill?	YES . . . . 1<br><br>NO 2	⟼21
20	How do you protect yourself from getting pregnancy	USE PILLS (SPECIFY) . . . . .1<br><br>ABSTENIENCE . . . . .2<br><br>CONDOMS . . . . 3<br><br>OTHERS (SPECIFY) 4	 <br>⟼27<br><br>⟼22<br><br>⟼27
21	What method or pills do you use	EC . . . . 1<br><br>OTHERS 2	
22	Where do you get this method/ pills	HOSPITAL . . . . 1<br><br>HEALTH CENTRE . . . . 2<br><br>COMMUNITY DISTRIBUTOR . . . . 3<br><br>FRIENDS . . . . 4<br><br>DON'T KNOW 5	IF CONDOM GO 24
23	What forced you to use EC	UNPROTECTED SEX 1	IF EC USED ASK

		OTHERS (SPECIFY) 2	
24	Who decided to use this method? You or your partner?	ME . . . . 1<br><br>PARTNER . . . . 2<br><br>BOTH 3	IF CONDOM ASK Q 26
25	What type of condom did you use?	FEMALE CONDOM . . . . .1<br><br>MALE CONDOM 2	IF CONDOM USED ASK
26	Are the EC available to you whenever you want to use?	YES . . . . .1<br><br>NO 2	
27	How long can it take for you to get EC?	30 MINUTES . . . . 1<br>1-2 HOURS . . . . 2<br><br>3-6 HOURS . . . . 3<br><br>OVER 6 HOURS 4	
**PART D: SOCIAL-CALTURAL PRACTICES**<br>**I WOULD LIKE TO ASK YOU ABOUT SOME THE ISSUES THAT HAPPENS WITHIN OUR COMMUNITIES**			
28	Have you ever heard of local medicine used to prevent a woman or a girl from getting pregnancy	HEARD . . . . 1<br><br>NOT HEARD 2	⟼30
29	Have you ever used the local medicine to protect you from getting pregnancy	YES . . . . 1<br><br>NO 2	
30	What cultural practices and beliefs performed here that prevent girls from using contraceptives	HERBS . . . . 1<br><br>BELIEF THAT CONTRACEPTIVES ARE FOR MARRIED WOMEN AND OLD PEOPLE. . . . . . 2<br><br>BELIEF THAT IF USE CONTRACEPTIVES BEFORE GIVNG BIRTH YOU WILL NEVER HAVE A CHILD . . . . 3<br><br>CONTRACEPTIVES CAUSES LONG MENSUARTION PERIOD . . . . .4<br><br>PEOPLE CONSIDERS YOU AS A PROSTITUTE 5	

		TOLD DURING INITIATION NEVER TO USE CONTRACEPTIVES .... 6<br><br>OTHERS (SPECIFY) 7	
31	What are social barriers that affect adolescents from getting contraceptives	LONG DISTANCES TO THE FACILITY .... 1<br><br>POVERTY ....2<br><br>NEED TO HAVE CHILD .... 3<br><br>MANTHA NDI MANYANZI .... 4<br><br>OTHERS (SPECIFY) 5	
**PART E: SEXUAL ACTIVITY IN THE PAST**<br><br>**I WOULD LIKE TO ASK YOU ABOUT YOUR SEXUAL LIFE IN THE PAST. PLEASE FEEL FREE TO ANSWER OR NOT**			
32	How old were you when you started experiencing menstrual periods?	10-14 (SPECIFY) .... 1<br><br>15-19 (SPECIFY) 2	
33	How old were you when you had first sexual intercourse	10-14 (SPECIFY) .... 1<br><br>15-19 (SPECIFY) 2	
34	When was the last time you had sex intercourse	NEVER HAD HAD .... 1<br>DAYS AGO .... 2<br>WEEKS AGO ....3<br>MONTHS AGO .... 4<br>YEARS AGO 5	
35	When you had last sexual intercourse did you do anything to protect you from getting pregnancy?	YES .... 1<br><br>NO 2	↦36
36	What did you use to protect you from getting pregnancy?	PILLS(SPECIFY) .... 1<br>HERBS ....2<br>CONDOMS .... 3<br>OTHERS 4	
37	Have you ever had pregnancy	YES .... 1<br>NO 2	↦39
38	What was the outcome of the pregnancy you had had?	INDUCED ABORTION ....,1<br>SPONTANEOUSLY ABORTION .... 2<br>DELIVERED LIVE BABY ....3<br>FRESH DEAD BABY ....4<br>MACERATED BABY 5	

39	Do you have a boy friend	YES . . . . 1<br>NO 2	
**PART F: WAYS OF IMPROVING EC SERVICES**<br><br>**PLEASE ASK THOSE EVER USED EC ONLY**			
40	Are you satisfied with the way EC are found?	YES . . . . 1<br><br>NO . . . . 2<br><br>DON'T KNOW. 3	 <br>⟼ Q40<br><br>→ GO TO Q41
41	How satisfied/unsatisfied are you?	JUST SATISFIED . . . . 1<br><br>MODERATE SATISFIED . . . .2<br><br>VERY SATISFIED . . . . .4<br>JUST UNSATISFIED . . . . 5<br>MODERATE UNSATISFIED . . . . 6<br>VERY UNSATISFIED 7	
42	Apart from your usual place (NAME THE PLACE) where else would you prefer EC should be found	SCHOOLS . . . . .1<br><br>PEERS . . . . .2<br><br>RETAILS . . . . 3<br><br>VILLAGES . . . . 4<br><br>OTHERS SPECIFY 5	CHECK Q22
43	Would you prefer to get EC from the following places? (PROBE FULLY)	SCHOOLS . . . . 1<br><br>PEERS . . . . 2<br><br>VILLAGE HEADS . . . . 3<br><br>HSAs . . . . .4<br><br>COMMUNITY DISTRIBUTORS . . . . 5<br><br>YOUTH CLUBS/CENTRES . . . . .6<br><br>OTHERS (SPECIFY) 7	
44	Who do you prefer should provide ECs	HSAs 1	

		COMMUNTY DISTRIBUTORS . . . . .2<br><br>PEERS . . . . .3<br><br>NURSES/DOCTORS . . . . 4<br><br>OTHERS SPECIFY 5	

THANK THE RESPONDENT AND END THE INTERVIEWS
