# Supplementary material for: School-going adolescent girls’ preferences and views of family planning services in Phalombe district, Malawi: A descriptive, cross-sectional study
Source: PLoS One. 2022 May 3;17(5):e0267603. doi: 10.1371/journal.pone.0267603 (PMC9064102; doi:10.1371/journal.pone.0267603)
Supplement: S2 Questionnaire — (PDF) [file pone.0267603.s002.pdf]

SCHOOL GOING ADOLESCENT GIRLS' PREFERENCE AND VIEWS OF FAMILY PLANNING SERVICES QUESTIONNIERE IN CHICHEWA

TIME STARTED:……………………………………

TIME ENDED……………………………………… **DATE:…………………………**

INTERVIEW NO …………………………………… **SERIAL NO …………………..**

NO	QUESTIONS	CODING CATE GORIES	SKIP
**PART A: SOCIAL DEMOGRPHIC OF THE RESPONDENTS<br>TSOPANO NDIKUFUNSA MAFUNSO OKUNZANA ZAMBIRI ZAINUYO**			
01	Muli ndi dzaka zakubadwa zingati?	10-14 (SPECIFY)…………………… …… 1<br>15-19 (SPECIFY) … ……………………….2	
02	Muli kalasi/ folomu yanji?	STANDARD 5-8(SPECIFY)…………………1<br>FORM 1-4 (SPECIFY) …………………… ..2	
03	Ndiinu a mpingo wanji?	MUSLIM …………………………………1<br>CATHOLIC ……………………………….2<br>CCAP …………..………………………..3<br>ANGLICAN ………………………………4<br>SEVETH DAY ADVENTIST/BAPTIST ……. 5<br>OTHERS CHRISTIAN ……………………. 6<br>OTHERS ………………………………… 7	
04	Ndiinu a mtundu wanji?	CHEWA …… ………………………….. 1<br>LOMWE ………………………………… 2<br>YAO ……………………………………. 3<br>MANG, ANJA …………………………… 4<br>OTHERS SPECIFY ………………………. 5	
**PART B-1: KNOWLEDGE ABOUT CONTRACEPTIVES**			
05	Kodi mudamvapo kapena mumaziwa zokhunzana ndi njila zolera za mtundu wina uliwonse?	YES ………..…………………………..1<br>NO …………………………………….. 2	→ Q7
06	Ndi njila zanji imene mudamvapo kapena imene mumaiziwa?	FEMALE STERILIZATION ……………… A<br>MALE STERILIZATION …………………B<br>IUD ……………………………………..C<br>INJECTABLES ………………………….D<br>IMPLANTS ……………………………..E<br>PILL ………. ……………………………F<br>MALE CONDOM .. ..…..…………………. G<br>FEMALE CONDOM ……………………..H<br>EMERGENCY CONTRACEPTION ………… I<br>STANDARD DAYS METHOD ……………. J<br>LACTATIONAL AMENORRHEA METHOD … K<br>RHYTHM METHOD …… ……………… .L<br>WITHDRAWAL ……..……………………M<br>OTHER MODERN METHOD ……………. N<br>OTHER TRADITIONAL METHOD ..……….. O	⟼ Q8
07	Nanga kodi mudamvapo za (PROBE EACH)?	FEMALE STERILIZATION ……………… A<br>MALE STERILIZATION ………………… B<br>IUD ……………………………………. C<br>INJECTABLES ………………………… D	

		IMPLANTS . . . . E<br>PILL . . . . .F<br>CONDOM . . . . .G<br>FEMALE CONDOM . . . . .H<br>STANDARD DAYS METHOD . . . . I<br>LACTATIONAL AMENORRHEA METHOD. . . .J<br>RHYTHM METHOD.. K<br>WITHDRAWAL . . . . L<br>OTHER MODERN METHOD . . . . .M<br>OTHER TRADITIONAL METHOD . . . . .N<br>EMERGENCY CONTRACEPTION . . . . .O	IF EC NOT MENTIONED GO Q 9
08	Mudamvera kuti za njila ya pangozi imene mkazi amatha kugwilitsa ntchito ngati atagona ndi mwamuna mosadzitetedza	GOVERNMENT FACILITIES . . . . 1<br>CHAM . . . . 2<br>PRIVATE HOSPITAL/CLINIC . . . . 3<br>BLM/PSI/GAIA................ . . . . 4<br>SCHOOL................................ . . . . .5<br>FRIENDS............................. . . . . 6<br>CHURCH.......................... . . . . 7<br>RADIO (SPECIFY).................... . . . . .8<br>TV (SPECIFY)....................................... . . . 9<br>OTHERS (SPECIFY)..................... 10	
09	Mukudziwa njila ina ili yonseyo imene mkazi angathe kugwilitsa ntchito atatha kugonana ndi mwamuna osazitetedza?	YES . . . . 1<br>NO 2	⟼Q11
10	Ndi njila yanji imeneyi?	ABSTENANCE . . . . 1<br>USING HERBS . . . . 2<br>PILLS (SPECIFY) . . . . .3<br>OTHERS (SPECIFY). 4	
11	Mumapanga chani pofuna kudzitetedza kuti musatenge pakati?	USING HERBS . . . . .1<br>ABSTENIENCE . . . . .2<br>CONTRACEPTIVES (specify). . . . . .3<br>OTHERS SPECIFY 4	IF EC MENTIONED ASK Q12 IF NOT GO Q13
12	Ndi nthawi iti imene mkazi angagwilitse ntchito njila yadzizizi yolera? ASK RESPONDENT TO SPECIFY	IMMEDIATELY AFTER SEX INTERCOUR. . . . . . . .1<br>HOURS BEFORE HAVING SEX INTERCOURSE. . . .2<br>FEW DAYS AFTER HAVING SEXUAL INTERCOIURSE . . . . .3<br>DON'T KNOW 4	
13	Inu mutafuna lero mungathe kupedza njila ya dzizizi yolera kuti musakhale ndi pakati?	YES . . . . .1<br>NO 2	⟼ PART B-2
14	Mungakapedze kuti njila ya dzizizi yolera (PROBE WHERE ELSE UNTIL NO MORE ANSWER)	GOVERNMENT FACILITIES . . . . .1<br>CHAM . . . . .2<br>PRIVATE HOSPITAL/CLINIC . . . . .3<br>BLM/PSI/GAIA................ . . . . .4<br>SCHOOL................................ . . . . 5<br>FRIENDS............................. 6	

		COMMUNITY DISTRIBITOR. . . . . 7<br>OTHERS (SPECIFY)…………………. 8	
	**PART B-2: KNOWLEDGE ABOUT FETILE PERIOD**<br><br>NOW LETS TALK ABOUT WHEN A WOMEN IS MORE LIKELY TO GET PREGNANCY. PLEASE FEEL FREE TO ANSWER OR NOT.		
15	Ndiliti limene munasamba kapena kupita kumwenzi komalidza?	DAYS AGO . . . . .1<br>WEEKS AGO . . . . .2<br>MONTHS AGO (SPECIFY) . . . . .3<br>NEVER HAD HAD PERIODS 4	⟼17<br>⟼17<br><br>⟼17
16	Mwanena kuti munasamba komalidza miyezi yapitayo chifukwa chani zili choncho?	AM PREGNANT . . . . 1<br>HAVING ABNORNAL PERIODS . . . . .2<br>USING HERBS . . . . 3<br>USING CONTRACEPTIVES . . . . 4<br>DON'T KNOW . . . . 5<br>OTHERS SPECIFY 6	⟼ CHECK Q 11
17	Kuyambira pamene mkazi wasamba kufikira msambo wina, kodi pali matsiku omwe mkazi angathe kutenga mimba mosabvuta ngati atakhala kuti wagonana ndi mwamuna mosazitetedza?	YES . . . . 1<br>NO . . . . .2<br>DON'T KNOW 3	⟼ GO TO C<br>⟼ GO TO C
18	Nthawi imeneyi ndi iti? Kodi atangosala pang,ono kuti ayambe nsambo, kapena mkatikati mwansambo kapena atangomalidza kumene nsambo kapena pakatikati pa misambo iwiri?	JUST BEFORE HER PERIODS BEGINS . . . . . . .1<br>DURING HER PERIODS . . . . 2<br>RIGHT AFTER HER PERIOD HAS ENDED . . . 3<br>HALF WAY BETWEEN TWO PERIODS 4	
	**PART C: AVAILABILITY**<br><br>**TSOPANO NDIKUFUNA NDIZIWE MENE MUMAZITETEDZERA KUTI MUSATENGE MIMBA**		
19	Pa miyedzi 6 yapitayi kodi mwagwilitsapo ntchito njila yolera ina ili yonseyo?	YES . . . . 1<br>NO 2	⟼21
20	Mumatani pofuna kuti mudzitetedze kuti musatenge pakati?	USE PILLS (specify). . . . . .1<br>ABSTENIENCE . . . . .2<br>CONDOMS . . . . 3<br>OTHERS (SPECIFY) 4	⟼27<br>⟼22<br>⟼27
21	Ndi njila/mapilisi anji amene munagwilitsa ntchito?	EC . . . . 1<br>OTHERS 2	
22	Munakapedza kuti njila imeneyi?	HOSPITAL . . . . 1<br>HEALTH CENTRE . . . . 2<br>COMMUNITY DISTRIBUTOR . . . . 3<br>FRIENDS . . . . 4<br>DON'T KNOW 5	IF CONDOM GO 24
23	Chinakupangitsani ndichani kuti mugwilitse ntchito njila ya kulera ya dzizizi	UNPROTECTED SEX . . . . 1<br>OTHERS (SPECIFY) 2	ASK IF EC USED

24	Ndindani anaganinza zoti mugwilitse njila imeneyi? Inuyo kapena wokondedwa wanu?	ME . . . . 1<br>PARTNER . . . . 2<br>BOTH 3	IF CONDOM ASK Q 26
25	Ndi mtundu wanji wa kondomu imene munagwilitsa ntchito?	FEMALE CONDOM . . . . .1<br>MALE CONDOM 2	IF CONDOM USED ASK
26	Kodi mapilitsi a kulera mwadzizizi amapedzeka mdela lanu lino nthawi ina ili yonse mungawafune kuti mugwilitse ntchito?	YES . . . . .1<br>NO 2	
27	Zingakutengeleni nthawi yayitali bwanji kuti muchoke kunyumba ndikukafika komwe mutha kupedza ma pilisi a njila yolera ya dzizizi?	30 MINS . . . . 1<br>1-2 HOURS . . . .2<br>3-6 HOURS. . . . . .3<br>OVER 6 HOURS 4	
**PART D: SOCIAL-CALTURAL PRACTICES**<br>**TSOPANO NDIKUFUNA KUKUFUTSANA ZA ZINTHU ZINA ZIMENE ZIMATHA KUCHITIKA MDELA AMENE TIMAKHALA**			
28	Kodi mudamvapo za mankhwala azitsamba amene akadzi amatha kugwilitsa ntchito pozitetedza kuti asatenge pakati?	HEARD . . . . 1<br>NOT HEARD 2	⟼30
29	Munayamba mwagwilitsapo ntchito mankhwala achikuda kuti akutetezeni kuti musakhale ndi pakati?	YES . . . . 1<br>NO 2	
30	Kodi ndi zikhalidwe kapena zikhulupiliro ziti zimene zimachitika mdela lanu ndipo zimapangitsa kuti amtsikana asamagwilitse ntchito njila za kulera za kuchipatala?	HERBS . . . . 1<br>BELIEF THAT CONTRACEPTIVES ARE FOR MARRIED WOMEN AND OLD PEOPLE. . . . . . 2<br>BELIEF THAT IF USE CONTRACEPTIVES BEFORE GIVNG BIRTH YOU WILL NEVER HAVE A CHILD . . . . 3<br><br>CONTRACEPTIVES CAUSES LONG MENSUARTION PERIOD . . . . 4<br>PEOPLE CONSIDERS YOU AS A PROSTITUTE . . . . .5<br>TOLD DURING INITIATION NEVER TO USE CONTRACEPTIVES . . . . 6<br>OTHERS (SPECIFY) 7	
31	Nanga ndizinthu ziti zimene zimalepheletsa kuti amtsikana azikanika kukatenga njila zolera za kuchipatala?	LONG DISTANCES TO THE FACILITY . . . . 1<br>POVERTY . . . . .2<br>NEED TO HAVE CHILD . . . . .3<br>FEAR AND SHYNESS . . . . .4<br>OTHERS (SPECIFY) 5	
**PART E: SEXUAL ACTIVITY IN THE PAST**<br><br>**PANO NDIKUFUNSENI ZOKHUNZANA NDI NKHANI ZOKUGONANA MBUYOMU. CHONDE KHALANI OMASUKA PONDIFOTOKONZERA BWINOBWINO**			
32	Munali ndi dzaka zingati pamene mumayamba kunsamba?	10-14 (SPECIFY) . . . . 1<br>15-19 (SPECIFY) . . . . 2<br>NEVER HAD HAD PERIODS 3	
33	Munali ndi dzaka zingati pamene munagonana ndi mwamuna koyamba	10-14 (SPECIFY) . . . . 1<br>15-19 (SPECIFY) . . . . 2<br>NEVER HAD HAD SEX 3	⟼39

34	Ndiliti limene munagonana ndi mwamuna komalidza?	NEVER . . . . 1<br>DAYS AGO . . . . 2<br>WEEKS AGO . . . . 3<br>MONTHS AGO . . . . 4<br>YEARS AGO 5	
35	Pa nthawi yomwe munagonana ndi mwamuna komalidza munagwilitsa ntchito njila ina ili yonse kuzitetedza kuti musatenge pakati?	YES . . . . 1<br>NO 2	⟼ 36
36	Munagwilitsa ntchito njila yanji pofuna kuzizteteza kuti musatenge pakati pamene mumagonana ndi mamuna komalidza?	PILL S (SPECIFY) . . . . 1<br>HERBS . . . . 2<br>CONDOMS . . . . 3<br>OTHERS 4	
37	Munayamba mwatenga pakati?	YES . . . . 1<br>NO 2	⟼39
38	Kodi pathupi pamenepa zotsatira zake zinali zotani?	INDUCED ABORTION . . . . 1<br>SPONTANEOUS ABORTION . . . . 2<br>LIVE BIRTH . . . . 3<br>FRESH DEAD BABY . . . . 4<br>MACERATED 5	
39	Muli ndi chibwenzi?	YES . . . . 1<br>NO 2	
**PART F: WAYS OF IMPROVING EC SERVICES**			
40	Ndiinu wokhutila ndi mene njila za kulera mwapangonzi zikupedzekera kuno?	YES . . . . 1<br>NO . . . . 2<br>DON'T KNOW. . . . 3	⟼ TO Q 42<br>→TO Q 42
41	Ndiinu wokhutila/wosakhutira motani?	JUST SATISFIED . . . . 1<br>MODERATE SATISFIED . . . . 2<br>VERY SATISFIED . . . . 3<br>JUST UNSATISFIED . . . . 4<br>MODERATE UNSATISFIED . . . . 5<br>VERY UNSATISFIED 6	
42	Kupatulako kumalo komwe mumapedza njila za kulera mwa pangozi nthawi zonse ndikuti kwina komwe mungakonde kuti zizipedzeka?	SCHOOLS . . . . 1<br>PEERS . . . . 2<br>RETAILS . . . . 3<br>VILLAGES . . . . 4<br>OTHERS SPECIFY 5	CHECK Q22
43	Mungakonde njila za kulera mwapangonzi zitamapedzeka malo awa (PROBE)	SCHOOLS . . . . 1<br>PEERS . . . . 2<br>VILLAGE HEADS . . . . 3<br>HSAs . . . . 4<br>COMMUNITY DISTRIBUTORS . . . . 5<br>YOUTH CLUBS/CENTRES . . . . 6<br>OTHERS (SPECIFY) 7	
44	Ndindani amene mukanakonda kuti azipedzeka ndikumapereka njila za kulera mwadzizizi?	HSAs . . . . 1<br>COMMUNTY DISTRIBUTORS . . . . 2<br>PEERS . . . . 3<br>NURSES/DOCTORS 4	

		OTHERS SPECIFY 5	

THANK THE RESPONDENT AND END THE INTERVIEWS
